# Pseudomonas putida and its close relatives: mixing and mastering the perfect tune for plants

**DOI:** 10.1007/s00253-022-11881-7

**Published:** 2022-04-30

**Authors:** Stefanie Bernardette Costa-Gutierrez, Conrado Adler, Manuel Espinosa-Urgel, Ricardo Ezequiel de Cristóbal

**Affiliations:** 1 Planta Piloto de Procesos Industriales Microbiológicos (PROIMI-CONICET), Avenida Belgrano Y Pasaje Caseros, 4000 San Miguel de Tucumán, Tucumán, Argentina; 2 Instituto Superior de Investigaciones Biológicas (INSIBIO, CONICET-UNT) E Instituto de Química Biológica “Dr. Bernabé Bloj”, Facultad de Bioquímica, Química y Farmacia, Universidad Nacional de Tucumán, 461, 4000 San Miguel de Tucumán, Chacabuco, Tucumán Argentina; 3 Department of Environmental Protection, Estación Experimental del Zaidín, CSIC, Profesor Albareda 1, 18008 Granada, Spain

**Keywords:** *Pseudomonas putida*, Plant growth–promoting rhizobacteria (PGPR), Abiotic stress, Biocontrol, Sustainable agriculture

## Abstract

**Abstract:**

Plant growth–promoting rhizobacteria (PGPR) are a group of microorganisms of utmost interest in agricultural biotechnology for their stimulatory and protective effects on plants. Among the various PGPR species, some *Pseudomonas putida* strains combine outstanding traits such as phytohormone synthesis, nutrient solubilization, adaptation to different stress conditions, and excellent root colonization ability. In this review, we summarize the state of the art and the most relevant findings related to *P. putida* and its close relatives as PGPR, and we have compiled a detailed list of *P. putida* sensu stricto, sensu lato, and close relative strains that have been studied for their plant growth–promoting characteristics. However, the mere in vitro analysis of these characteristics does not guarantee correct plant performance under in vivo or field conditions. Therefore, the importance of studying adhesion and survival in the rhizosphere, as well as responses to environmental factors, is emphasized. Although numerous strains of this species have shown good performance in field trials, their use in commercial products is still very limited. Thus, we also analyze the opportunities and challenges related to the formulation and application of bioproducts based on these bacteria.

**Key points:**

•*The mini-review updates the knowledge on Pseudomonas putida as a PGPR.*

•* Some rhizosphere strains are able to improve plant growth under stress conditions.*

•* The metabolic versatility of this species encourages the development of a bioproduct.*

## Introduction

The uncontrolled use of chemical fertilizers and pesticides to increase crop yields is of concern in terms of environmental deterioration, wildlife conservation, and human health. Simultaneously, due to inadequate land management and different environmental factors, soil degradation has intensified through drought, flooding, high temperatures, and soil salinity. An environmentally friendly alternative that can address these issues is the use of biofertilizers as plant growth–promoting rhizobacteria (PGPR) (Basu et al. 2021). PGPRs are free-living bacteria that can enhance plant growth and/or provide protection against biotic or abiotic stresses by colonizing roots (Kloepper and Schroth 1978). These microorganisms have long been considered a promising tool, but their mechanisms of action and performance under real field conditions are still a matter of research.

Bacteria shown to enhance plant growth belong to different genera, such as *Azospirillum* (Coniglio et al. 2019), *Azotobacter* (El-Nahrawy and Yassin 2020), *Bacillus* (Kashyap et al. 2019), *Rhizobium* (Al-Karhi et al. 2019), *Serratia* (Singh and Jha 2016), and *Klebsiella* (Bhardwaj et al. 2017). Nevertheless, *Pseudomonas* is considered one of the most promising groups as potential biofertilizers, due to their numerous plant growth–promoting (PGP) traits (Nadeem et al. 2016). *Pseudomonas* is a genus of aerobic, Gram-negative, rod-shaped, polar flagellated bacteria containing over 200 species and countless strains. Fluorescent Pseudomonads are capable of synthesizing water-soluble yellow-green fluorescent siderophores, which is a very valuable characteristic from the taxonomic point of view. It is a ubiquitous genus, with an astonishing metabolic diversity that allows it to colonize a wide range of ecological niches and adapt to marginal environments. Some bacteria belonging to this genus have been isolated from polluted environments and are also common inhabitants of aquatic environments and the rhizosphere. *Pseudomonas* species are frequently found as free-living epiphytic rhizobacteria, although some can also colonize the root interior as endophytes (Andreolli et al. 2021). Due to their ubiquity and physiological and genetic diversity, this group of bacteria is of great ecological importance.

The taxonomic classification of *Pseudomonas* species has long been contentious, due to the lack of conserved phenotypic differences. Recently, *P. putida* KT2440 (accession number AE015451), the best characterized member of this group, has been proposed for reclassification as *P. alloputida* KT2440 (cluster Pp5) (Keshavarz-Tohid et al. 2019). Other well-known *P. putida* strains were also re-classified into *P. alloputida* including BIRD-1, F1, and DOT-T1E. This re-classification as members of a novel species is based on the fact that the mentioned strains are distant from the type strain *P. putida* NBRC 14164 T 55, but has still not been fully accepted in the scientific community, and in fact, these strains remain as *P. putida* in all standard databases (such as NCBI and Pseudomonas Genome Database).

The best studied *Pseudomonas* PGPR strains include the *P. fluorescens* complex (including *P. protegens*, *P. chlororaphis*, *P. brassicacearum*, and *P. koreensis*) (Ashraf et al. 2019; Kang et al. 2021; Wang et al. 2020a, b; Zhang et al. 2020), *P. stutzeri* (Lami et al. 2020), and *P. putida* (Costa-Gutierrez et al. 2020a; 2020b). Despite the large number of reviews about *Pseudomonas* strains (e.g., Bhimeshwari et al. 2018; Nadeem et al. 2016; Shaikh et al. 2020), the role of *P. putida* as a plant growth promoter has been neglected in the literature. The present review contributes to vindicate this species as a source for new bioproducts and to fill the knowledge gap on this topic.

This review provides, in a concise and holistic manner, the most relevant insights about the direct and indirect mechanisms by which *P. putida* strains enhance plant growth under stressed and non-stressed conditions. In addition, it focuses on how *P. putida* can colonize and persist in the rhizosphere. Finally, a brief update is provided on the prospects and limitations of the use of *P. putida* as biofertilizer and formulations for their commercialization.

## Plant growth promotion activities and traits

The field of bacterial PGP activities is so vast, and many excellent review articles cover the topic thoroughly (e.g., Goswami et al. 2016; Vejan et al. 2016; Mehmood et al. 2018); therefore, it will not be reviewed here. Instead, this review focuses on the role of *P. putida* strains as plant growth promoters. Sometimes, incomplete taxonomic analysis or species assignment based on phenotypic characteristics leads to rather limited and sometimes confusing information about this species regarding its PGPR characteristics. Therefore, a detailed list of *P. putida* sensu stricto (species assignment based on genome sequence), sensu lato (species assignment based on 16S rDNA sequence), and close relatives of *P. putida* (strains identified as *P. putida* by biochemical tests, Biolog, or FAME profile) that have shown PGP characteristics can be found in Table 1.

**Table 1:** Selected examples of *Pseudomonas putida* sensu stricto, sensu lato, and close relatives as plant growth promoting bacteria

Strains	Isolation location	Plant species tested	Experimental conditions^d^	Effects on plant	PGPR traits				References
					*Indole compounds*	*Siderophore synthesis*	*Phosphate solubilization*	*ACC deaminase*	
**Sensu stricto**^**a**^									
KT2440^e^ (accession number AE015451)	Soil from a vegetable orchard. Japan	Corn, soybean, and *Arabidopsis thaliana*	In vitro and in vivo; normal, salt, and heat stress conditions	Increase germination rates, root and shoot lengths, fresh and dry weights; ISR					Arslan and Akkaya (2020), Costa-Gutierrez et al. (2020a; (2020b), Molina-Romero et al. (2017), Nelson et al. (2002), Planchamp et al. (2015)
MTCC5279^e^ (accession number AMZE00000000)	Desert regions	*A. thaliana* and chickpea	In vitro and greenhouse; salt stress	Increase plant growth; ISR		NR^f^		NR^f^	Chaudhry et al. (2013), Srivastava et al. (2012), Tiwari et al. (2016)
BIRD-1^e^ (accession number CP002290)	Garden soil	Corn, cucumber, zucchini, lettuce, chard, pepper, melon, onion, tomato, and bean	In vitro, microcosm, and greenhouse	Increase germination rates and height of plants				NR^f^	Matilla et al. (2011a), Roca et al. (2013)
**Sensu lato**^**b**^									
FBKV2 (accession number KT311002.1)	Rhizosphere eggplant	Corn	Normal and drought stress conditions	Increase root and shoot lengths, and dry weight					Vurukonda et al. (2016)
GN04 (accession number KF282767)	Heavy metal and hydrocarbon contaminated soil. Trinidad, Casanare, Colombia	Corn	Greenhouse. Normal and copper contaminated soils	Increase plant growth and chlorophyll content; protection from copper toxicity				ND	Rojas-Tapias et al. (2014)
PAN2 (accession number HM590706)	Arbuscular mycorrhiza associated bacteria. *Glomus mosseae* spores from different guava cropping systems. Southern India	Guava	Polythene bags with a mixture of garden soil, farmyard manure, and sand	Increase root, shoot, leaf and stem dry weights, total leaf area, and total biomass		NR		NR	Panneerselvam et al. (2012)
AKMP7 (accession number GU396282)	Rhizosphere of sorghum grown under semiarid conditions. India	Wheat and *A. thaliana*	Pots; normal and heat stress conditions; no effect under water stress condition	Increase root and shoot lengths, dry weight, and grain formation				NR	Ali et al. (2011), Shah et al. (2017)
CR7 (accession number AY785244)	Rhizosphere of corn	Corn	Greenhouse and field	Increase root and shoot dry weights; biocontrol		NR		NR	Mehnaz et al. (2010)
Rs-198 (accession number FJ788425)	Alkaline soil	Cotton and pepper	Greenhouse and field; normal and salt stress conditions	Increase plant height, germination rate, seedling growth, fresh and dry weights; protection against salt stress		NR			He et al. (2016; (2019), Yao et al. (2010)
GAP-P45 (accession number GQ221267)	Rhizosphere of sunflower	Sunflower, corn, and *A. thaliana*	In vitro; salt, water, and drought stress conditions	Increase survival, plant biomass, and root adhering soil/root tissue ratio of seedlings				NR	Ghosh et al. (2018), Sandhya et al. (2009; (2010a; (2010b)
CC-FR2-4 (accession number DQ193603)	Rhizosphere of *Ficus religiosa* L	Lettuce	Gnotobiotic	Increase root and shoot lengths				NR	Rekha et al. (2007)
B0 (accession number MTCC 6842)	Sub-Alpine Location. Indian Central Himalaya	Corn	Greenhouse	Increase plant biomass; biocontrol against fungus	NR			NR	Pandey et al. (2006)
CQ179 (accession number AY958233)	Rhizosphere of corn	Corn	Greenhouse	Increase root and shoot lengths; biocontrol		NR		NR	Mehnaz and Lazarovits (2006)
KNP9 (accession number DQ205427)	Panki Power plant. India	Mung bean	Greenhouse	Increase root and shoot growth	NR		NR	NR	Tripathi et al. (2005)
UW4^e^ (accession numbers CP003880 and NC_019670)	Rhizosphere of reeds	Canola, cucumber, *Brassica campestris*, *Pinus pinaster*, tomato, and wheat	Gnotobiotic, in vitro; normal and salt stress and cold conditions	Increase root and shoot lengths, shoot fresh and dry weights; biocontrol		NR^f^	NR^f^		Cheng et al. (2007; (2012), Duan et al. (2013), Gamalero et al. (2010), Glick et al. 1995), Hao et al. (2007), Nascimento et al. (2013), Tabatabaei et al. (2016), Yan et al. (2014)
**Close relatives**^**c**^									
53/5	Rhizosphere	Tea	Field	Increase plant growth		NR	ND		(Çakmakçı (2016)
4 and 108	Bacterial Culture Collection of Soil and Water Research Institute (SWRI)	Corn	Field; drought stress	Protection against drought stress			NR		Ansary et al. (2012)
W2	Rhizosphere of wheat	Wheat	Jar and pots; normal and salt stress conditions	Increase root and shoot lengths, and seedling biomass		NR			Nadeem et al. (2010)
Wp1 Cfp10, Wp150 and Wp159	Rhizosphere of wheat and canola. Iran	Wheat and canola	Field	Increase plant height, root length and crop yield					Abbas-Zadeh et al. (2010)
B29/2	Rhizosphere of tea	Strawberries	Greenhouse and field; normal and water stress conditions	Increase plant growth and yield. Physiological and biochemical changes		NR			Çakmakçi (2016), Çakmakçi et al. (2010), Erdogan et al. (2016)
N21	Rhizosphere of wheat	Wheat	Pots; salt stress	Increase plant height, root length, and grain yield	NR	NR			Zahir et al. (2009)
TSAU 1	Rhizosphere of wheat in saline soil	Wheat	Normal and salt stress conditions	Increase root and shoot lengths		NR	NR		Egamberdieva and Kucharova (2009)
Biovar B HS-2	Soil at nickel-contaminated sites	Canola	Greenhouse, pots with soil contaminated with nickel	Increase plant biomass and nickel uptake by shoots and roots					Rodriguez et al. (2008)
Spp	Rhizosphere of pea	Pea	Drought stress	Increase shoot length, flowering pod formation and grain yield		NR			Arshad et al. (2008)
Subgroup B strain 1	Rhizosphere of tomato	Tomato	Greenhouse	Increase root and shoot lengths and weights; biocontrol		NR	ND		Gravel et al. (2007)
RC-06	Rhizosphere of wheat	Barley, wheat, spinach, and strawberries	Greenhouse and field; normal and water stress conditions	Increase root and shoot weights		NR			Çakmakçi et al. (2006; (2007a; (2007b), Erdogan et al. (2016)
Biotype A	Rhizosphere of corn	Corn	Gnotobiotic	Increase plant height, root weight, and total biomass		NR	NR		Shaharoona et al. (2006)

*ACC* 1-aminocyclopropane-1-carboxylate, *ISR* induced systemic resistance, *ND* not detected, *NR* not reported, detected

^a^Species assignment based on genome sequence

^b^Species assignment based on 16S rDNA sequence

^c^Strains identified as *P. putida* by biochemical tests, Biolog, or FAME profile

^d^Unless specified, the setup was under regular conditions (i.e., no stress applied)

^e^Complete genome sequence available

^f^Genes were detected for this activity

### Availability of nutrients for plant uptake

Nitrogen is an essential element for all forms of life. It is required for the synthesis of nucleic acids, enzymes, proteins, and chlorophyll II. Although atmospheric nitrogen constitutes 78% of the air, this gaseous form of the element cannot be taken by plants. However, some bacteria are able to metabolize nitrogen and reduce it to a plant-assimilable form, such as ammonia (NH_3_), by means of the complex enzymatic system nitrogenase. Iron is essential for nitrogen-fixing microorganisms as a component of Fe- and MoFe-proteins of nitrogenase. Two types of biological nitrogen fixation (BNF) can be distinguished: symbiotic and non-symbiotic. In the former, there is a mutualistic relationship between plant and bacteria that allows the formation of nodules in which BNF occurs. In the latter, nitrogen fixation is carried out by non-symbiotic bacteria, such as *Pseudomonas* spp., without plant association (Noreen et al. 2019). Since *P. putida* strains seem to be unable to fix nitrogen naturally, an approach involving engineering bacteria with recombinant DNA was used to render this species a nitrogen fixer. That was the case of *P. putida* KT2440 carrying the *nif* gene from the donor strain *P. stutzeri* A1501 (Setten et al. 2013). However, its use as a PGPR is currently not possible, as the release of genetically modified bacteria into the environment is not accepted.

Phosphorus, together with nitrogen, is a highly required element for plant nutrition. Phosphorus is involved in metabolic processes such as photosynthesis, energy transfer, signal transduction, macromolecule biosynthesis, and respiration. This element is abundantly available in the soil, both as organic and inorganic compounds. Nonetheless, phosphorus is not directly assimilable by plants from these compounds since these are insoluble, immobilized, or precipitated forms of phosphorus. Plant-assimilable soluble forms are mono- and di-basic phosphate (H_2_PO_4_^−^ and HPO_4_^2−^, respectively). Some bacteria can solubilize phosphorus to plant-assimilable forms by different strategies, such as the production of organic or inorganic acids and mineralization by phosphatases. Table 1 displays several examples of *P. putida* strains capable of solubilizing phosphorus. Many *Pseudomonas* strains solubilize inorganic phosphate by producing extracellular organic acids such as gluconic and 2-ketogluconic acids (Miller et al. 2010; Oteino et al. 2015). The regulation of gluconic acid-production mechanisms was deciphered in *P. putida* KT2440 (An and Moe 2016).

Analysis of the *P. putida* BIRD-1 genome revealed that it encodes at least five phosphatases related to phosphorus solubilization, one of them being a phytase. Phytases facilitate the mineralization of the main form of organic phosphorus in soil (phytate) (Roca et al. 2013). Recently, two novel phytase-encoding genes (*ppp1* and *ppp2*) have been identified and characterized in *P. putida* strain P13 (Sarikhani et al. 2019). In the genome of *P. putida* KT2440, the phytase gene (*appA*) is not annotated in the sequence; however, engineered strains showed phytase activity and increased plant growth in mung bean and *Arabidopsis thaliana* (Patel et al. 2010; Shulse et al. 2019). Engineered *P. putida* strains overexpressing *appA* could be a promising tool for rendering phytate-phosphorus (P) available to plants and promoting their growth.

### Phytohormone production and modulation

Phytohormones are endogenous bioactive organic substances synthesized by plants, which are involved in various plant growth processes. Five main phytohormones can be distinguished: auxins, gibberellins, cytokinins, abscisic acid, and ethylene. As detailed below, certain PGPRs have been shown to produce some of these molecules or to modulate their synthesis by the plant, thus altering its physiology.

Indole acetic acid (IAA) is the most common phytohormone belonging to the auxin group and plays a major role in the development of the plant root system. However, IAA levels above some threshold value (specific for each plant) inhibit root growth (Duca et al. 2018). Several IAA biosynthetic pathways have been described according to their intermediates being tryptophan the most studied IAA precursor. Spaepen et al. (2007) provide a comprehensive overview of bacterial IAA biosynthesis pathways. In general, phytopathogenic bacteria, such as *Agrobacterium tumefaciencs* and *P. syringae* pathovars, synthesize IAA via tryptophan through the intermediate indoleacetamide. In contrast, beneficial bacteria, such *P. putida* strains, produce IAA mainly by via indole-3-pyruvic acid, an alternative tryptophan-dependent pathway. In the genome of the plant growth–promoting rhizobacterium, *P. putida* BIRD-1, many PGP traits were found, including an overproduction of IAA through convergent pathways (Matilla et al. 2011a; Roca et al. 2013). Some examples of *P. putida* strains with reported IAA synthesis are displayed in Table 1.

Both gibberellins and cytokinins play an important role in plant physiological processes, as protein synthesis regulation, chlorophyll accumulation, seed germination, stems and shoot elongations, and cell division. Abscisic acid production is stimulated during abiotic stresses, such as drought, salinity, or extreme temperatures. Reports on the production of gibberellins, cytokinins, and abscisic acid by *P. putida* strains are scarce. The synthesis of gibberellin by *P. putida* strains has been associated with abiotic stress tolerance in plants. For example, *P. putida* H-2–3 synthesizes gibberellin and modulates stress and hormonal physiology in soybean, improving plant growth under salinity and drought conditions (Kang et al. 2014), and *P. putida* Rs-198, which exhibits high levels of IAA and gibberellin production, increased cotton biomass under salinity conditions (He et al. 2016). The role of gibberellins under saline conditions is also associated with mitigating the deleterious effects of salt stress by increasing water availability to plants (Colebrook et al. 2014). Recently, it was reported that inoculation of rice plants with *P. putida* KT2440 stimulates an alternative plant defense mechanism based on abscisic acid accumulation (Wang et al. 2020a, b).

Ethylene is a gaseous phytohormone related to fruit ripening and induces physiological changes in plants. It is also known as a stress hormone. Under stress conditions, such as drought, salinity, and pathogenicity, ethylene production increases affecting plant growth. The enzyme 1-aminocyclopropane-1-carboxylate (ACC) synthase is involved in ethylene synthesis. ACC deaminase activity can reduce the amount of ACC (immediate precursor of ethylene) and thus reduce ethylene levels, improving plant development and protecting against environmental stress. The presence of the ACC-deaminase enzyme has been reported in a number of *P. putida* strains (see Table 1) and makes this species a promising bioinoculant for promoting plant growth under different types of environmental stresses. For example, *Pseudomonas* sp*.* UW4 (a close relative of *P. putida*) increased tomato tolerance to flooding stress (Grichko and Glick 2001); promoted canola plant growth at low temperature under salt stress (Cheng et al. 2007; 2012); stimulated cucumber plant growth under salt stress (Gamalero et al. 2010); protected tomato plants against salt stress and increased shoot length, shoot fresh, and dry mass and chlorophyll concentration through synergy between ACC deaminase activity and trehalose (Del Carmen Orozco-Mosqueda et al. 2019; Yan et al. 2014); ACC deaminase activity has also been involved in the biocontrol of pine trees against the nematode *Bursaphelenchus xylophilus* (Nascimento et al. 2013). Inoculation of ACC deaminase-producing *P putida* could address the problem of salinity in agricultural soils.

### Biocontrol

Pathogenic organisms, such as fungi, bacteria, viruses, and insects, are responsible for significantly reducing crop yields, causing global economic losses annually. The biocontrol activities of *P. putida* strains are diverse and are well documented (Weller 2007). Some mechanisms are as follows: competition for space; fluorescent *Pseudomonas* have high growth rates that added to their abilities to adapt to diverse environmental conditions and allow them to compete with pathogens; competition for nutrients, such as iron through the synthesis of siderophores (Daura-Pich et al. 2020; Saritha et al. 2015); antibiosis (Sun et al. 2017); mechanisms of secretion of toxic compounds such as bacterial type VI secretion systems (T6SSs) (Bernal et al. 2017); chitinolytic activity; production of ammonia, hydrogen cyanide, protease, and urease (Saritha et al. 2015); and induced systemic resistance (ISR) (Matilla et al. 2009; Meziane et al. 2005).

ISR can be defined as the physiological state of plants in which their defense capacity is enhanced in response to a specific environmental stimulus, and as a result, the innate defense of the plant is increased against a wide variety of pathogens. In general, ISR is mediated by salicylic acid, jasmonic acid, and ethylene pathways (Kamle et al. 2020). These pathways are involved in *P. putida* PCI2 during the defense of tomato plants against *Fusarium oxysporum* MR193 (Pastor et al. 2016). The role of siderophores in ISR has also been reported in *P. putida* WCS358 (currently classified as *Pseudomonas* sp. WCS358) during the defense of *Eucalyptus urophylla* against *Ralstonia solanacearum* (Ran et al. 2005). This strain can activate ISR in *A. thaliana*, tomato, and bean against *P. syringae* pv. *tomato* and *F. oxysporum* f. sp. *raphani* (Meziane et al. 2005; Van Wees et al. 1997). Interestingly, ISR in *Pseudomonas* sp*.* WCS358 involves flagella, pseudobactin, and lipopolysaccharide as complementary rather than additive compounds, since mutants in any of the aforementioned compounds were able to trigger the ISR response similarly to the wild-type strain (Meziane et al. 2005). It seems that not all plant species are susceptible to the biocontrol mechanisms of *Pseudomonas* sp*.* WCS358, since the ISR response could not be triggered in carnation and radish (Duijff et al. 1993; Leeman et al. 1995; Meziane et al. 2005). *P. putida* KT2440 also triggered ISR response against *Colletotrichum graminicola* in corn (Planchamp et al. 2015). In this strain, haem peroxidase seems to be essential for ISR activation in *A. thaliana* (Matilla et al. 2009), and benzoxazinoids synthesis may induce the bacterial production of ISR-eliciting compounds (Neal and Ton 2013).

An effective strategy to control the wide range of soil pathogens on agronomically important species is to take advantage of symbiotic associations between arbuscular mycorrhizal (AM) fungi and *P. putida* strains (Panneerselvam et al. 2012; 2013). *P. putida* strains jointly with AM fungi showed antagonistic potential against soil borne pathogens, such as *F. oxysporum*, *Ceratocystis fimbriata*, and *Sclerotium rolfsii* (Saritha et al. 2015) and the nematode *Meloidogyne incognita* in chickpea (Akhtar and Siddiqui 2007). On the other hand, the application of a co-culture of two *Pseudomonas* sp. strains (WCS358 and RE8) with different disease-suppressive mechanisms enhanced biocontrol activity in radish against *F. oxysporum*, compared to single-strain treatments (De Boer et al. 2003). This increase in biocontrol activity could be due to the combined use of bacteria with different biocontrol mechanisms, e.g., *Pseudomonas* sp. WCS358 can compete for iron by siderophore synthesis, while *Pseudomonas* sp. RE8 can trigger ISR (De Boer et al. 2003).

### Siderophores

Under iron-limiting conditions, such as in bulk soil or rhizosphere, microbes produce siderophores to scavenge the essential metal and thus favor niche colonization. Siderophore production and uptake has long been recognized as a relevant trait in PGPRs. Several siderophore-producing strains of *P. putida* are shown in Table 1. *P. putida*, as many other Pseudomonads, produces the siderophore pyoverdine which has three distinctive elements: a quinoline-1-carboxylic acid moiety responsible for the green fluorescence observed in all pyoverdine variants, a dicarboxylic acid or its monoamide bound to the 5-amino group of the chromophore, and a peptide chain having 6 to 14 amino acids bound to the carboxylic group of the quinoline (Barrientos-Moreno et al. 2019; Schalk et al. 2020). Pyoverdine variants, most resulting from differences in peptide chains, have been identified at the species level by isoelectric focusing. The method was termed siderotyping and serves as a taxonomic tool (Ye et al. 2013). However, this method may have limitations in identifying some strains within this species. For example, an isoelectric focusing analysis revealed that the pyoverdine of the strain *P. putida* KT2440 and G4R is identical (Matthijs et al. 2009). Compared to other Pseudomonads, the more diverse structure of pyoverdines within *P. putida* species allowed strain-level characterization based on the correlation of siderotypes and phylogeny of genes required for pyoverdine production (Meyer et al. 2008; Ye et al. 2013). This diversity of siderotypes is further accompanied by specific outer membrane receptors (FpvA) for each pyoverdine variant (Ye et al. 2013). Thus, it has been proposed that receptors and modular NRPS enzymes involved in siderophore synthesis co-evolved (Bodilis et al. 2009; Smith et al. 2005).

Pyoverdine, produced by Pseudomonads capable of colonizing plant roots, has been shown to facilitate iron uptake by plants in different model systems (e.g., *A. thaliana*, tomato, pea, clover, and grasses) (Lurthy et al. 2020; Nagata et al. 2013; Trapet et al. 2016). As mentioned previously, siderophore production is involved in biological control against pathogens, e.g., biological control of the pathoghen *Xanthomonas fragariae* by *P. putida* KT2440 was reported to require pyoverdin (Henry et al. 2016). In *P. putida* B2017, pyoverdin synthesis is also involved in biocontrol activity against *F. oxysporum* f.sp. *radicis-lycopersici* in tomato, *Rhizoctonia solani* and *Pectobacterium atrosepticum* in potato, and *Sclerotinia sclerotiorum* in lettuce (Daura-Pich et al. 2020; Oliver et al. 2019).

## Colonization and persistence in the rhizosphere

Soon after the first descriptions of plant growth–promoting or biocontrol *Pseudomonas* strains, it became apparent that detection of PGP activities in vitro was not sufficient to ensure a positive influence on plant growth, even under controlled conditions. The ability of the bacteria to efficiently establish and persist in the rhizosphere environment proved to be key in showing their beneficial effect (Amaya-Gómez et al. 2020). This led to a significant amount of research on the genetic and environmental factors that determine colonization efficiency. While most of the early work focused on long-term studies, much less attention was given to the early stages of interaction between bacteria and plant roots, which may be essential for successful root colonization and persistence.

### Adhesion to plant surfaces and biofilm formation

Pioneering work was done using random transposon mutagenesis to identify *P. putida* functions required for adhesion to seeds, as an initial step for further establishment on plant roots (Espinosa-Urgel et al. 2000). In fact, the initial phase of colonization by *P. putida* seems to be very active, with the bacterial population relative to root biomass reaching its maximum 24–48 h after seedling inoculation (Espinosa-Urgel et al. 2002). Beyond this period, the growth of the root-associated bacterial population is coupled to the development of the plant; thus, the number of bacteria recovered per root weight remains basically stable afterward.

Different studies have shown that some genetic elements involved in attachment to seeds and roots of plants are also involved in attachment to abiotic surfaces and biofilm formation (Espinosa-Urgel et al. 2000; Nielsen et al. 2011; Nilsson et al. 2011; Yousef-Coronado et al. 2008). However, both processes do not completely overlap, and some functions required for efficient establishment on plant surfaces do not seem to be relevant on abiotic surfaces, while others are essential in both cases. Perhaps the best characterized elements are the adhesins LapA and LapF, the two largest proteins of *P. putida*, with over 8000 and 6000 amino acids, respectively. These proteins show a repetitive structure and translocate to the bacterial surface through dedicated Type I secretion systems (Hinsa et al. 2003; Martínez-Gil et al. 2010). They have a sequential role in biofilm development, with LapA being involved in cell-to-surface attachment and LapF in cell-to-cell interactions, respectively (Martínez-Gil et al. 2010), although both are likely to be part of the extracellular matrix of mature biofilms. Mutations in either protein, or in the elements required for their secretion, decrease biofilm formation, reduce seed attachment, and hamper competitive root colonization in corn plants (Hinsa et al. 2003; Martínez-Gil et al. 2010; Yousef-Coronado et al. 2008). Their importance, however, may vary depending on plant species and environmental conditions. The same is true for the different exopolysaccharides (EPS) produced by *P. putida*: cellulose (Bcs), alginate, and two species-specific EPS, Pea and Peb. Although mutants in any of the operons encoding these elements show reduced fitness in the rhizosphere, in some reports, Bcs appears as the main contributor to survival, while in others, alginate and Pea are described as the most relevant (Martínez-Gil et al. 2013; Nilsson et al. 2011). Alginate plays a specific role for survival and biofilm formation under water stress conditions (Chang et al. 2007), but overproduction of other EPS takes place in alginate-deficient mutants (Nielsen et al. 2011). Similarly, the lack of LapA and/or LapF causes increased expression of the *pea* operon, leading to EPS overproduction, whereas EPS mutants generally show reduced expression of the two adhesins (Martínez-Gil et al. 2013).

All these data suggest that the structural elements involved in root colonization by *P. putida* establish complex modulatory connections. It seems likely that environmental cues determine the balance between these elements, so that the biofilm matrix composition and/or attachment mechanism adjust to the existing conditions. However, the regulatory network that modulates such balance remains to be fully understood.

### Environmental factors affecting root colonization

Different environmental factors and chemical signals influence biofilm formation. Among them, the availability of carbon and energy sources in the medium determines the multicellular behavior of many species. However, a detailed exploration of the influence of metabolic signals or how specific nitrogen, carbon, and energy sources impact biofilm formation and root colonization by *P. putida* has yet to be performed. This information could be relevant for the optimization of its use as PGPR.

Numerous evidences have demonstrated that iron is another key element in bacterial multicellular behaviors, and iron limitation has recently been reported to be a relevant factor in antagonistic interactions between rhizosphere microorganisms (Eng et al. 2020). Competition for iron is an important factor in the rhizosphere, and the ability of *P. putida* and other *Pseudomonas* to efficiently transport iron complexed to siderophores produced by other microorganisms is one of the key strategies for successful displacing competitors (Fernández-Piñar et al. 2011; Mirleau et al. 2000). Iron present in corn seeds is important for their colonization by *P. putida* KT2440 (Molina et al. 2005), and mutations affecting iron acquisition lead to reduced fitness in the rhizosphere (Molina et al. 2005; 2006). Furthermore, pyoverdine-mediated iron acquisition is required for swarming motility (Matilla et al. 2007). Although as mentioned above, siderophore production has long been known as a relevant trait in PGPRs, the wide number of additional iron capture systems that *P. putida* can employ makes it difficult to ascertain their specific role in each environmental situation.

Calcium is also known to regulate adhesion processes in a wide range of bacteria. In *P. putida*, calcium seems to alter the normal kinetics of biofilm formation, promoting early attachment and early detachment, whereas the calcium chelator EGTA causes a decrease in biofilm formation at concentrations that do not affect planktonic growth (Martínez-Gil et al. 2012). The effect of calcium may be, at least in part, through LapF, since the C-terminal domain of this protein (containing putative Ca^+2^ binding sites) was shown to form large aggregates in the presence of calcium that dispersed when EGTA was added (Martínez-Gil et al. 2012). However, the influence of calcium in the specific context of establishing root-associated populations has not been explored, even though tri-calcium phosphate or calcium phytate are commonly used to test phosphate solubilization activity of PGPRs.

### Motile versus biofilm populations in the rhizosphere

In the analysis of genetic determinants involved in seed attachment and root colonization by *P. putida*, mutants defective in flagellar motility were identified, but their fitness in the root system was not as affected as in the case of, for example, *lapA* mutants, suggesting that biofilm formation would be more relevant than motility (Yousef-Coronado et al. 2008). However, in other fluorescent *Pseudomonas* flagellar, motility had been reported as a key function for root colonization (Martínez-Granero et al. 2006). This apparent contradiction seems to derive mostly from the methodology used to analyze root-associated populations, in some cases taking into account the whole root system while in others considering only the root tip. In essence, this seems to reflect the fact that different subpopulations exist in the rhizosphere depending on the local environment. This idea is supported by the differential localization of wild-type and flagella-deficient strains of *P. putida* on corn roots (Yousef-Coronado et al. 2008), and the preferential colonization of older parts of the root by hyperadherent derivatives (Matilla et al. 2011b). Swarming motility, rather than swimming motility, has been proposed to drive root tip colonization by *P. putida* during plant growth (Matilla et al. 2011b). This movement along the root surface is likely linked to a chemotactic response to specific molecules released in areas where exudation is higher.

### Chemotaxis

Motile microorganisms are able to sense clear and consistent chemical gradients in the environment and to actively move toward or away from specific chemical sources. This phenomenon, known as chemotaxis, has been thoroughly studied in *Escherichia coli*; however, several studies have been performed in *P. putida.* Chemical signals can act as chemoattractants or chemorepellents. Although the list of chemoattractants is extensive, few chemorepellent molecules have been identified so far. *P. putida* strains have been shown to be attracted to a wide range of growth substrates, such as aromatic compounds, amino acids, and tricarboxylic acid cycle intermediates (Parales et al. 2004). This species is an excellent model for bioremediation, as they are attracted to aromatic hydrocarbons such as naphthalene and toluene (Lacal et al. 2011). In fact, two chemotactic phenotypes toward toluene were observed in *P. putida* strains, while strains KT2440 and F1 exhibited a moderate taxis in which bacteria approach at a distance of 1–2 mm, strain DOT-T1E showed a closer approach (strong chemotaxis or hyperchemotaxis) (Lacal et al. 2011). To our knowledge, no chemorepellent has been studied in *P. putida.* For more details on the chemosensory system and signaling pathway of *Pseudomonas*, we refer the reader to Sampedro et al. (2015) and references therein.

In the case of root-colonizing species, such as *P. putida*, active chemotaxis toward root exudates is a decisive process to ensure successful colonization in plant roots. The chemical composition of root exudates is dynamic and comprises a myriad of compounds such as sugars, organic acids, amino acids, fatty acids, and flavonoids. Given the broad metabolic repertoire of *P. putida* strains, the ability to chemotactically respond to several molecules present in root exudates is an expected trait. In fact, to date, 27 different chemoreceptors have been identified in *P. putida* KT2440. Chemotaxis of *P. putida* toward roots was mainly studied using corn plants as a model, confirming positive chemotaxis of *P. putida* KT2440 toward benzoxazinoids like DIMBOA and DIBOA (López-Farfán et al. 2019; Neal et al. 2012). Studies revealed that the transcription of chemoreceptor genes is highly dependent on the concentration of corn root exudates, increasing at low concentrations and generally decreasing at high concentrations of root exudates. This indicates that chemotaxis is likely greater at a distance, but decreases in the root vicinity, where other bacterial mechanisms may ensure root colonization (López-Farfán et al. 2019).

## Bioformulations

As it has been pointed out in this review, *P. putida* strains exhibit numerous characteristics that make them promising PGPRs, and some of them have their genome completely sequenced and available (see Table 1). Nonetheless, formulations based on this remarkable species are still scarce in the market. To date, the only commercial product that the authors could find is Fosfogel® (Bio-Iliberis R&D), based on *P. putida* BIRD-1. The use of this bioinoculant promotes rooting and plant growth, mainly due to IAA synthesis and phosphatase activity related to phosphorous solubilization.

Some improvements are being made regarding the optimization of low-cost culture media, industrial formulations, and large-scale cultivation of *P. putida* strains. For example, the culture medium for strain Rs-198 was optimized providing a basis for industrialized fermentation of the IAA-producing strain (Peng et al. 2014); also, the bacteria were successfully immobilized in Ca-alginate-bentonite-starch microcapsules which increased their survival and colonization rates in cotton roots and also increased the production of IAA and gibberellins compared to free cells under both saline and non-saline conditions (He et al. 2016); strain *P. putida* A (ATCC 12,633) was also immobilized in Ca-alginate-perlite enhancing rhizosphere colonization and plant growth promotion of *A. thaliana* compared to free-living suspensions (Liffourrena and Lucchesi 2018), and strain B2017, which does not produce antibiotics or toxic compounds, was grown on a large scale (125 L bioreactors), making it a promising biocontrol product formulated with *P. putida* (Daura-Pich et al. 2020; Oliver et al. 2019). The aforementioned works represent a good approach for the development of a *P. putida*-based product for its application in agriculture.

## Future perspectives and concluding remarks

Crop production is facing unprecedented challenges; the transition from current agricultural practices to a more sustainable but efficient production model is one of the greatest challenges of twenty-first century. This goal can be achieved through biotechnological techniques. The application of plant growth promoting microorganisms has proven to be a greener approach suitable to improve cultivation of several plant species even under stressful environmental conditions such as drought, salinity, and high temperatures. The genus *Pseudomonas* has been extensively studied as PGPR microorganisms. In the present review, emphasis was placed on *P. putida* species due to its apparent innocuousness in terms of pathogenicity unlike other members of this genus such as *P. aeruginosa* and *P. syringae*. This environmentally friendly species is an excellent candidate for the development of inoculants to replace the use of chemical fertilizers, ensuring sustainable agriculture in the future. However, suggesting the complete elimination of chemical fertilizers may be a rather ambitious goal. Alternatively, the combination of a *P. putida*-based inoculant combined with a reduced amount of the recommended doses of chemical fertilizer may be suggested. In this way, the bioinoculant acts as a biostimulator rather than a biofertilizer. Recently, inoculation of *Pseudomonas* sp. strain P3-57 (accession number MK503664) jointly with 70% of chemical fertilizer improved cucumber quality and sensory traits. Interestingly, the authors declared that inoculation of the bacteria alone did not improve cucumber yields (Kafi et al. 2021). Therefore, it might be worthwhile to test *P. putida* strains with well-known PGP traits in combination with different doses of chemical fertilizers.

In the present review, the numerous PGP traits of *P. putida* strains have been thoroughly described. Most of the plant growth promotion trials were conducted under in vitro or controlled conditions, and there are very little examples of good performance of *P. putida* strains under real field condition. This phenomenon could be explained mainly by flaws during inoculation, low inoculum concentration, or weak adhesion or colonization of the roots or rhizosphere. Besides, for successful root/rhizosphere colonization and plant growth promotion, bacteria should be able to tolerate the environmental conditions. Therefore, it is recommended that plant growth promotion trials be conducted using the soil types and crops in which the bacteria will be used (Costa-Gutierrez et al. 2021). Another interesting approach is the combination of *P. putida* strains with other microorganisms in a consortium, which could exhibit better performance compared to the use of single microorganisms, due to the combination of complementary activities. This strategy would require further research to rationally design mixtures of microorganisms with no negative interaction and that enhance synergetic effects among consortium members. The use of *P. putida* jointly with other microorganisms for plant protection and growth promotion is an intriguing topic, although it has not been extensively developed in this review.

*P. putida* is a promising candidate for industrial production, as it can withstand stressful conditions on an industrial scale. There are numerous studies about the optimization of culture media using low-cost carbon source (including palm oil sludge and biodiesel-derived crude glycerol) and large-scale production of these bacteria, issues that are not addressed in this review but are of great importance when developing a bioinoculant. On the other hand, there are few reports on *P. putida*-based bioproduct formulation in the literature. As mentioned above, some reports highlighted the microencapsulation in calcium as an effective technology to immobilize *P. putida* cells for formulations. A profitable technique could be to microencapsulate other strains of this species, with known PGP abilities, and test their performance under field conditions. Based on that, a gigantic effort is needed to make large-scale bioinoculants a reality to support and uplift agricultural sustainability globally.
